# A Wavelet Relational Fuzzy *C*-Means Algorithm for 2D Gel Image Segmentation

**DOI:** 10.1155/2013/430516

**Published:** 2013-09-24

**Authors:** Shaheera Rashwan, Mohamed Talaat Faheem, Amany Sarhan, Bayumy A. B. Youssef

**Affiliations:** ^1^Informatics Research Institute, City for Scientific Research and Technological Applications, Borg El Arab, Alexandria, Egypt; ^2^Computers and Control Engineering Department, Faculty of Engineering, University of Tanta, Tanta, Egypt

## Abstract

One of the most famous algorithms that appeared in the area of image segmentation is the Fuzzy *C*-Means (FCM) algorithm. This algorithm has been used in many applications such as data analysis, pattern recognition, and image segmentation. It has the advantages of producing high quality segmentation compared to the other available algorithms. Many modifications have been made to the algorithm to improve its segmentation quality. The proposed segmentation algorithm in this paper is based on the Fuzzy *C*-Means algorithm adding the relational fuzzy notion and the wavelet transform to it so as to enhance its performance especially in the area of 2D gel images. Both proposed modifications aim to minimize the oversegmentation error incurred by previous algorithms. The experimental results of comparing both the Fuzzy *C*-Means (FCM) and the Wavelet Fuzzy *C*-Means (WFCM) to the proposed algorithm on real 2D gel images acquired from human leukemias, HL-60 cell lines, and fetal alcohol syndrome (FAS) demonstrate the improvement achieved by the proposed algorithm in overcoming the segmentation error. In addition, we investigate the effect of denoising on the three algorithms. This investigation proves that denoising the 2D gel image before segmentation can improve (in most of the cases) the quality of the segmentation.

## 1. Introduction

Two-dimensional gel electrophoresis (2D gel) allows separation of mixtures of proteins due to differences in their isoelectric points (pI), in the first dimension, and subsequently by their molecular weight (MWt), in the second dimension. Other techniques for protein separation exist, but currently 2D gel provides the highest resolution allowing thousands of proteins to be separated. The great advantage of this technique is that it allows, from very small amounts of material, the investigation of the protein expression for thousands of proteins simultaneously [1].

In this paper, one of the issues and challenges related to digital image analysis of the 2D gel images will be addressed, namely, the segmentation of the images. This segmentation is very crucial in such images as it is used to conclude the existence (or not) of malicious cells in the patient's protein sample. Image segmentation is defined as the process of dividing images into regions according to its characteristic, for example, color and objects present in the images. The result of image segmentation is in the form of images that are more meaningful, easier to understand, and easier to analyze. Correct segmented results are very useful for the analysis, predication, and diagnoses [2–5].

Fuzzy *C*-Means (FCM) is a method of clustering which allows one piece of data to belong to two or more clusters. This method (developed by Dunn in 1973 [4] and improved by Bezdek in 1981 [6]) is frequently used in pattern recognition. It also has been in image analysis processes such as segmentation [2–5, 7–11].

Clustering involves the task of dividing data points into homogeneous classes or clusters so that items in the same class are as similar as possible and items in different classes are as dissimilar as possible. Clustering can also be thought of as a form of data compression, where a large number of samples are converted into a small number of representative prototypes or clusters. Depending on the data and the application, different types of similarity measures may be used to identify classes, where the similarity measure controls how the clusters are formed. Some examples of values that can be used as similarity measures include distance, connectivity, and intensity [3, 4, 7].

In nonfuzzy or hard clustering, data is divided into crisp clusters, where each data point belongs to exactly one cluster. In fuzzy clustering, the data points can belong to more than one cluster, and associated with each of the points are membership grades that indicate the degree to which the data points belong to the different clusters [2, 8].

However, one disadvantage of standard FCM is that it does not consider any spatial information in image context, which makes it very sensitive to noise and other imaging artifacts. Recently, many researchers have incorporated local spatial information into the conventional FCM algorithm to improve the performance of image segmentation [5, 8, 9]. Some research studies developed a Sugeno-type rule-based system that imposed spatial constraints in order to enhance the results of fuzzy clustering.

Others modified the FCM objective function by including a spatial penalty on the membership functions which leads to an iterative algorithm that is very similar to the conventional FCM and allows the estimation of spatially smooth membership functions. An FCM_S algorithm was introduced by modifying the objective function of FCM to compensate for the gray (intensity) inhomogeneity and to allow the labeling of a pixel to be influenced by the labels in its immediate neighborhood [5, 9, 11]. Variants of this algorithm were introduced, called FCM_S1 and FCM_S2. Both of them replaced the neighborhood term of FCM_S by computing in advance the extra mean-filtered image and median-filtered image, respectively.

An interesting modification of the FCM algorithm was to apply a wavelet function to the image before starting to segment it. In [10], Noreen et al. applied the Discrete Wavelet Transform (DWT) to MRI image to extract high level details and after some processing on this high pass image, they added it to the original image to get a sharpened image. The wavelet transformed image is then segmented by Fuzzy *C*-Means algorithm followed by Kirch's line/edge detection mask to further enhance the edge detail in the image. The wavelet transform has been also applied prior to other segmentation techniques like Watershed as in [12]. In both cases the application of wavelet greatly improved the quality of segmentation.

In our work, we propose an algorithm that makes use of wavelets and FCM and adds the relation notion to it in order to obtain better segmentation results. Through wavelets we extract the high pass image. The noise-robust nature of wavelets and the noise sensitivity of FCM combined in our algorithm ensure giving better segmentation results. The proposed algorithm focuses on the solution of over- and undersegmentation problem of low contrast images by applying preprocessing to the input image. The algorithm will be applied here to 2D gel images; however, it can be applied to any type of images.

This paper is organized as follows. This section gives the introduction to the area of interest of the research; then the basics of the Fuzzy *C*-Means algorithm and the fuzzy relation are introduced in Section 2. The details of the proposed algorithm are given in Section 3. The parameters of the algorithm are obtained experimentally in Section 4.  Section 5 demonstrates the experimental results on real 2D gel images, as well as comparisons with the FCM and the wavelet FCM.  Section 6 investigates performing denoising on the image before segmentation on the quality of the proposed segmentation algorithm. Finally, conclusions and future perspectives of this work are discussed in Section 7.

## 2. Background and Related Work

### 2.1. Fuzzy *C*-Means Algorithm

Fuzzy clustering belongs to the group of soft computing techniques which is often better suited for the real applications and there is very often no sharp boundary between clusters [13]. In fuzzy clustering we use membership values, instead of crisp values, which range between zero and one. 

The resulting data partition improves data understanding and reveals its internal structure. Partition clustering algorithms divide up a dataset into clusters, where similar data objects are assigned to the same cluster, whereas dissimilar data objects should belong to different clusters [4, 13].

The standard Fuzzy *C*-Means uses the Euclidean distance as a cost function to be minimized and expressed as

(1) $$\begin{array}{c} J_{m}=\sum_{i=1}^{N}\sum_{j=1}^{C}u_{ij}^{m}{||x_{i}-c_{j}||}^{2},\;1\leq m<\infty , \end{array}$$

where *m* is any real number greater than 1, *u*_*ij*_ is the degree of membership of *x*_*i*_ in the cluster *j*, *x*_*i*_ is the *i*th of d-dimensional measured data, *c*_*j*_ is the d-dimensional center of the cluster, and ||∗|| is any norm expressing the similarity between any measured data and the center [3, 8, 10].

In the standard use of Fuzzy *C*-Means, the weighting coefficient (*m*) is set to *m* = 2. The FCM algorithm runs iteratively in the following steps.

**Step 1**

Initialize *U* = [*u*_*ij*_] matrix, *U*(0).

**Step 2**

At *k*-step, calculate the centers vectors *C*(*k*) = [*c*_*j*_] with *U*(*k*)

(2) $$\begin{array}{c} c_{j}=\frac{\sum_{i=1}^{N}u_{ij}^{m}\cdot x_{i}}{\sum_{i=1}^{N}u_{ij}^{m}}. \end{array}$$

**Step 3**

Update *U*(*k*), *U*(*k* + 1)

(3) $$\begin{array}{c} u_{ij}=\frac{1}{\sum_{k=1}^{C}{\left(||x_{i}-c_{j}||/||x_{j}-c_{R}||\right)}^{2/\left(m-1\right)}}. \end{array}$$

**Step 4**

If ||*U*(*k* + 1) − *U*(*k*)|| < threshold, then

   STOP;

 otherwise return to Step 2.

The computation of the updated membership function is the condition for the minimization of the objective function. With Fuzzy *C-*Means, the centroid of a cluster is computed as being the mean of all points, weighted by their degree of belonging to the cluster [3, 8, 10].

The degree of being in a certain cluster is related to the inverse of the distance to the cluster. By iteratively updating the cluster centers and the membership grades for each data point, FCM iteratively moves the cluster centers to the “right” location within a dataset.

### 2.2. Fuzzy Relations

A Fuzzy relation [14] generalizes classical relation into one that allows partial membership and describes a relationship that holds between two or more objects.

Let *X* and *Y* be nonempty sets. A fuzzy relation *R* is a fuzzy subset of *X* × *Y*. In other words, *R* ∈ *F*(*X* × *Y*). If *X* = *Y*, then we say that *R* is a binary fuzzy relation in *X*. A fuzzy relation *R* is a relation that measures the degree by which *X* is related to *Y*.

Let *R* be a binary fuzzy relation on *R*. Then *R*(*u*, *v*) is interpreted as the degree of membership of the ordered pair (*u*, *v*) in *R*.

As a demonstrating example, let *R* be a binary fuzzy relation on *U* = {1,2, 3}, called “approximately equal,” and can be defined as

(4) R(1,1)=R(2,2)=R(3,3)=1,R(1,2)=R(2,1)=R(2,3)=R(3,2)=0.8,R(1,3)=R(3,1)=0.3.

The membership function of *R* is given by

(5) $$\begin{array}{c} R\left(u,v\right)=\{\begin{array}{cc} 1 & \text{if}\;u=v,\\ 0.8 & \text{if}\;\left|u-v\right|=1,\\ 0.3 & \text{if}\;\left|u-v\right|=2. \end{array} \end{array}$$

### 2.3. Wavelet Transform

Wavelets [15] are mathematical functions that allocate data into different frequency components and then study each component with a resolution matched to its scale. They have advantages over traditional Fourier methods in analyzing physical situations where the signal contains discontinuities and sharp spikes.

The wavelet transform has become a useful computational tool for a variety of signal and image processing applications. For example, the wavelet transform is useful for the compression of digital image files; smaller files are important for storing images using less memory and for transmitting images faster and more reliably.

There are two basic types of wavelet transform. One type of wavelet transform is designed to be easily reversible (invertible); that means the original signal can be easily recovered after it has been transformed. This kind of wavelet transform is used for image compression and cleaning (noise and blur reduction). The second type of wavelet transform is designed for signal analysis, for example, to detect faults in machinery from sensor measurements, to study biomedical signals or filter medical images, and to determine how the frequency content of a signal evolves over time. In these cases, a modified form of the original signal is not needed and the wavelet transform need not be inverted. In our work, we will use the second type to enhance and filter the image before executing segmentation. There are a number of ways of defining a wavelet (or a wavelet family) through scaling filter, scaling function, and wavelet function. An orthogonal wavelet is entirely defined by the scaling filter—a low-pass finite impulse response (FIR) filter of length 2*N* and sum 1. In biorthogonal wavelets, separate decomposition and reconstruction filters are defined. Also, wavelets are defined by the wavelet function *ψ*(*t*) (i.e., the mother wavelet) and scaling function *φ*(*t*) (also called father wavelet) in the time domain. Moreover, the wavelet only has a time domain representation as the wavelet function *ψ*(*t*). We can divide the wavelets according *t* the wavelet function to: continuous wavelet transform and discrete wavelet transform. A continuous wavelet transform (CWT) is used to divide a continuous-time function into wavelets. Unlike Fourier transform, the continuous wavelet transform possesses the ability to construct a time-frequency representation of a signal that offers very good time and frequency localization.

A discrete wavelet transform (DWT), for example, “Haar” wavelet used in our algorithm, is any wavelet transform for which the wavelets are discretely sampled. As with other wavelet transforms, a key advantage it has over Fourier transforms is temporal resolution: it captures both frequency *and* location information (location in time). The DWT of a signal *x* is calculated by passing it through a series of filters. First the samples are passed through a low pass filter with impulse response *g* resulting in a convolution of the two:

(6) $$\begin{array}{c} y\left[n\right]=\left(x*g\right)\left[n\right]=\sum_{k=-\infty }^{\infty }x\left[k\right]g\left[n-k\right]. \end{array}$$

The signal is also decomposed simultaneously using a high-pass filter *h*. The outputs give the detail coefficients (from the high-pass filter) and approximation coefficients (from the low-pass filter).

## 3. The Proposed Wavelet Relational Fuzzy *C*-Means Algorithm

The proposed algorithm is a version of the well-known Fuzzy *C*-Means clustering algorithm. It builds on the conventional Fuzzy *C*-Means algorithm and introduces the notion of fuzzy relations to it in order to efficiently differentiate spot pixels from the varying background. The proposed algorithm has several phases.

The first phase of the proposed algorithm is to use a wavelet function on the image before performing the segmentation to enhance its quality. We extract the high level details for the image (four subbands (LL, LH, HL, and HH)). Then we set the approximation coefficients in LL equal to zero and apply inverse wavelet transform to obtain a high pass image (L1). Subsequently, we add L1 to the original image to get a sharpened image. This will help filtering the small irrelevant pixels that should not be confused with the spots found in the image, thus minimizing the oversegmentation error. (The oversegmentation error is to consider a non spot as a spot which increases the number of detected spots in the image leading to erroneous quantification results).

**Figure 1:**
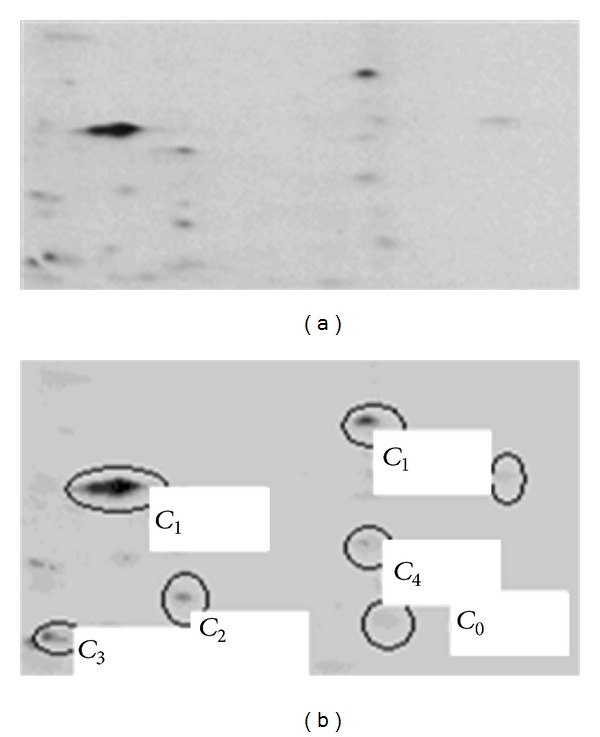
Part of 2D gel electrophoresis image when number of clusters (*C*) = 6.

Second, we use the conventional Fuzzy *C*-Means algorithm to obtain initial set of clusters. The only modification made to the FCM algorithm is setting the number of clusters (*C*) to more than two clusters, which was taken to be exactly two in the usual usage of the FCM algorithm. This setting is intended so as not to ignore the detection of the lighter protein spots from the background. A simple application of such an assumption is shown in Figure 1. From the figure, we can observe that even the lighter protein spots were detected at cluster *C*_4_.

However, this is not sufficient to detect proteins spots and quantify them accurately. A more robust method must exist to differentiate protein spots from the background varying gel rather than increasing the number of clusters only. This reveals the need of introducing the notion of fuzzy relations into the FCM algorithm which will be third phase of the proposed algorithm following the application of the FCM algorithm. 

We create a fuzzy relation *R*(*x*, *y*) between each two pixels (*x*, *y*) belonging to different clusters produced by the FCM. This fuzzy relation will define the degree of closeness between these pixels. Then, for each two pixels (*x*, *y*), we compare *R*(*x*, *y*) (which is the absolute value of the difference between the gray values of pixels *x* and *y*) to *β*, where *β* is a linguistic variable representing the fuzzy value “High.” Now if *R*(*x*, *y*) is “High,” then the pixel (*x* or *y*) representing the highest gray value is a spot pixel.

**Figure 2:**
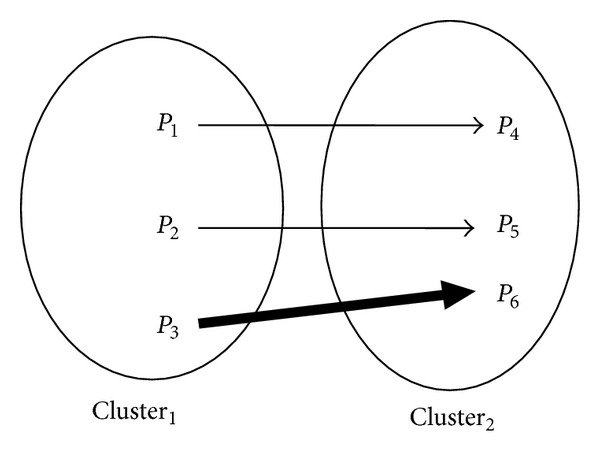
Representation of the fuzzy relation between pixels in two clusters.

Figure 2 shows a representation of a fuzzy relation, *R*(*x*, *y*), between two points (*x*, *y*) in two different clusters: Cluster_1_ and Cluster_2_. The arrows represent the degree of closeness between two pixels from different clusters. The dark arrow marks a strong closeness between the two pixels while the light arrow marks a weak closeness.

As a total, the proposed algorithm is composed of 7 steps, where the first three steps represent the first phase which uses the wavelet decomposition concept to filter the image before applying the FCM algorithm. The fourth step represents the second phase which applies the FCM to the produced image with number of clusters more than 2, to produce preliminary clusters. Finally, these clusters are internally refined to identify the inner spots by separating the background pixel from the contained pixel in the cluster using the fuzzy relational concept at steps (5) to (7) (third phase). A summary of the steps of the proposed algorithm is given in Algorithm 1.

The proposed algorithm with the above added features has the following advantages.

1. The number of spots detected in the image is increased by increasing the number of clusters to which the pixels in the image must be partitioned.
2. The problem of the collected protein spots in the same area will be solved since we put into consideration the luminance of the pixels in the image (i.e., degree of intensity). In this case, the quantification of protein spots will be much easier.
3. In the proposed algorithm, we do not care about the neighborhood pixels when investigating if the pixel is a spot pixel or not. Rather, we consider the pixels in different clusters. This means that even very small spots can be detected as long as they belong to a cluster which will solve the problem of the missing value.

## 4. Choice of Algorithm Parameters

### 4.1. Fuzzification of Parameter (*β*)

**Figure 3:**
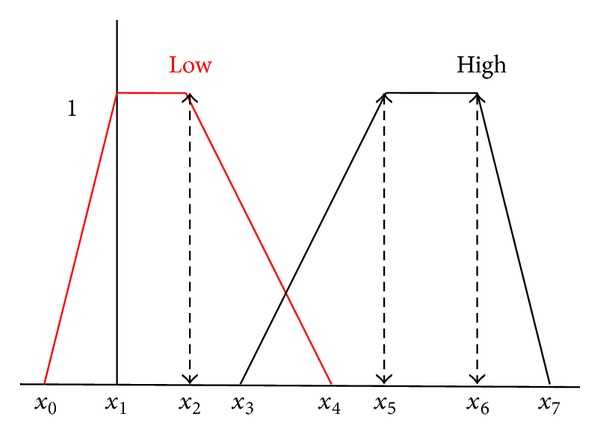
Trapezoidal membership function.

The performance of the proposed WRFCM algorithm relies greatly on two main parameters: *C* = number of clusters and *β* = degree of closeness between the two pixels. The determination of the proper value of parameter (*β*), which represents the threshold between the differences in the intensity of any two pixels in different clusters, is very important and critical. In order to estimate it, we used the trapezoidal fuzzy function [13] shown in Figure 3.

To illustrate the need for such function, let *k* represents the absolute difference between the two pixels *x* and *y*. If *k* is high, then max⁡(*x*, *y*) is a spot pixel. While if *k* is low and one of them is a spot pixel, then the other is a spot pixel. Now the objective is to define the membership functions low and high of this function.

The membership function low will be defined by four points (*x*_0_, *x*_1_, *x*_2_, *x*_4_). However, in order to have a real trapezoid, we need four points at the left of *x*_1_. Following the same reasoning, the membership function high will be defined by four points (*x*_3_, *x*_5_, *x*_6_, *x*_7_) (*x*_7_ any positive point > *x*_6_, being *x*_6_ the highest possible value for *x*). In case when the membership function is trapezoid (or pseudotrapezoid which in this case will be “low” and “high”), the membership function can be defined as

(7) ylow(x;x0,x1,x2,x4)  =max⁡(min⁡(x−x0x1−x0,1,x4−xx4−x2),0),yhigh(x;x3,x5,x6,x7)  =max⁡(min⁡(x−x3x5−x3,1,x7−xx7−x6),0).

### 4.2. Determining the Parameters by Software

The proper determination of the two parameters, number of clusters (*C*) and degree of closeness between the two pixels (*β*), ensures obtaining optimal or near-optimal performance of the algorithm. Both values will be computed experimentally. We performed the experiments on a data sample of human leukemia (a 2D gel image) with variable values of both *C* and *β* as shown in Figure 3. We use the evaluation method *F*, which indicates the average squared error proposed [3], to determine the performance of the algorithm at each test case. The objective quantitative evaluation function (*F*) for image segmentation is computed as

(8) $$\begin{array}{c} F\left(I\right)=\sqrt{N}\sum_{j=1}^{N}\frac{e_{j}^{2}}{\sqrt{S_{j}}}, \end{array}$$

where *N* is the number of regions in the segmented image and *S*_*j*_ is the area of region *j* (measured by number of pixels in this region *j*). We use *C*_*x*_(*p*) to denote the value of component *x* for pixel *p*. We define the average value of component *x* in region *j* by

(9) $$\begin{array}{c} C_{x}\left(R_{j}\right)=\frac{\left(\sum_{p\in R_{j}}C_{x}\left(p\right)\right)}{S_{j}}. \end{array}$$

The squared color error of region *j* (*R*_*j*_) is defined as

(10) $$\begin{array}{c} e_{x}^{2}\left(R_{j}\right)={\sum_{p\in R_{j}}\left(C_{x}\left(p\right)-C_{x}\left(R_{j}\right)\right)}^{2}. \end{array}$$

For any segmentation *I* in which the color error is zero for all segments (i.e., there is no variance in color within each region), the value of *F*(*I*) = 0 and hence a segmentation in which each pixel is in its own region will minimize the value of *F*.

For a complex image in which all cannot be zero, except for a segmentation in which each pixel is its own region, still *F* has two strong biases: segmentations with lots of regions are heavily penalized by $\sqrt{N}$, and segmentations that have regions with large areas are heavily penalized unless the large region is very uniform in color, since the total error (not average error) is used and only divided by the square root of the area of the region (versus being divided by *S*_*j*_, which would give the average squared error).

**Figure 4:**
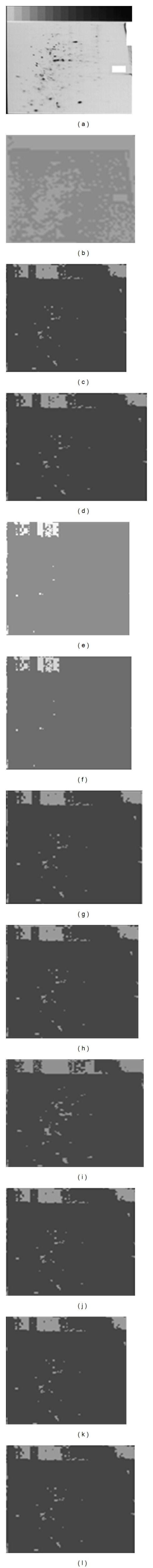
2D gel electrophoresis image of sample of patient-human leukemia: (a) original image, (b) gradient image, (c) gradient image after applying WRFCM when *C* = 4, *β* = 20, (d) gradient image when *C* = 6, *β* = 20, (e) gradient image when *C* = 8, *β* = 20, (f) gradient image when *C* = 10, *β* = 20, (g) gradient image when *C* = 4, *β* = 19, (h) gradient image when *C* = 4, *β* = 21, (i) gradient image when *C* = 4, *β* = 22, (j) gradient image when *C* = 4, *β* = 23, (k) gradient image when *C* = 4, *β* = 24, and (l) gradient image when *C* = 4, *β* = 25.

We have used the sample image of patient-human leukemia [16] to obtain the proper values of *β* and *C*. We have experimentally changed both values in the algorithm to choose the values that give the least possible error as shown in Figure 4.

Part of the experimental results is listed in Table 1. The proper values of *β* used in the membership function, shown in Figure 3, can be concluded from these results as follows: when the value of *β* ≤ 21, *β* ≥ 23, the error is low, while when *β* is 22, the error is high. So, we will take *β* to be 19.

The proper value of *C* accompanied with the proper value of *β* is 4. It is worth notifying that when *C* = 6, 8, and 10, the algorithm gave slightly better results; however, it consumed much more time as seen in Table 1 that is why we have chosen *C* = 4.

## 5. Software Results

In this section, we aim to evaluate the performance of the proposed algorithm against the conventional FCM algorithm and the Wavelet Fuzzy *C*-Means (WFCM) proposed by Noreen et al. [10]. We will use seven data samples chosen from the dataset for human leukemias (Eric Lester, Peter Lemkin), HL-60 cell lines (Eric Lester, Peter Lemkin), and fetal-alcohol-syndrome- (FAS-) serum (James Myrick, Mary Robinson, Peter Lemkin) in [16] to show the effectiveness of the proposed algorithm. The dataset is composed of four types of experiments with over 300 gif images with annotation and landmark data in html, tab-delimited, and xml formats. 2D gel images used in experiments are of resolution 512 × 512 pixels and are grayscale images.

To judge the performance of the algorithm, we will use two different methods: the visual acceptance of the segmented image and the numerical values that measure segmentation error *F* and also the quality metric PSNR which we introduce in this section to evaluate the segmentation. This quality metric is defined as follows [17]:

(11) $$\begin{array}{c} \text{PSNR}=20\cdot \log_{10}\left(\frac{\text{MAXI}}{\sqrt{\text{MSE}}}\right), \end{array}$$

where MAXI is the maximum possible pixel value of the image. When the pixels are represented using 8 bits per sample, MAXI = 255, and the mean squared error (MSE) for two *m* × *n* images *I* and *K* is defined as

(12) $$\begin{array}{c} \text{MSE}=\frac{1}{m_{}n}\sum_{i=0}^{m-1}\sum_{j=0}^{n-1}{\left[I\left(i,j\right)-K\left(i,j\right)\right]}^{2}. \end{array}$$

A higher PSNR would normally indicate that the segmentation is of higher quality.

**Figure 5:**
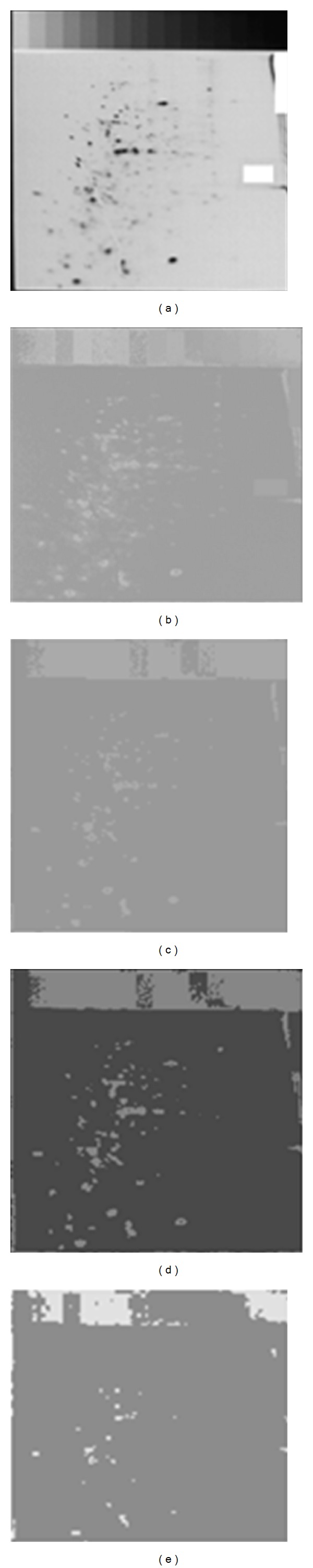
2D gel electrophoresis image of the first sample of patient-human leukemias: (a) original image, (b) gradient image, (c) gradient image after applying FCM segmentation algorithm, (d) gradient image after applying WFCM segmentation algorithm, and (e) gradient image after WRFCM segmentation algorithm.

The visual results of applying the conventional Fuzzy *C*-Means segmentation algorithm on one of these images (2D gel electrophoresis image of the first sample of Patient-Human Leukemias) at *C* = 2, the results of applying the Wavelet Fuzzy *C*-Means (WFCM) proposed, and the results of applying the proposed Wavelet Relational Fuzzy *C*-Means (WRFCM) algorithm at *C* = 4 and *β* = 19 are shown in Figure 5. All the implementations in this section had been performed using MATLAB 7.3.

From the results shown in Figure 5, we can conclude that the proposed algorithm (WRFCM) achieves high performance and detects the protein spots more precisely, as shown in Figure 5(e); even the less dark spots in the image appear. In Figure 5(c) when applying the FCM algorithm, those protein spots disappeared totally which affects the spot quantization step in the whole process of 2D gel image analysis, whereas the WFCM algorithm suffered from oversegmentation error by detecting more spots than what exist in the original images (Figure 5(d)).

**Figure 6:**
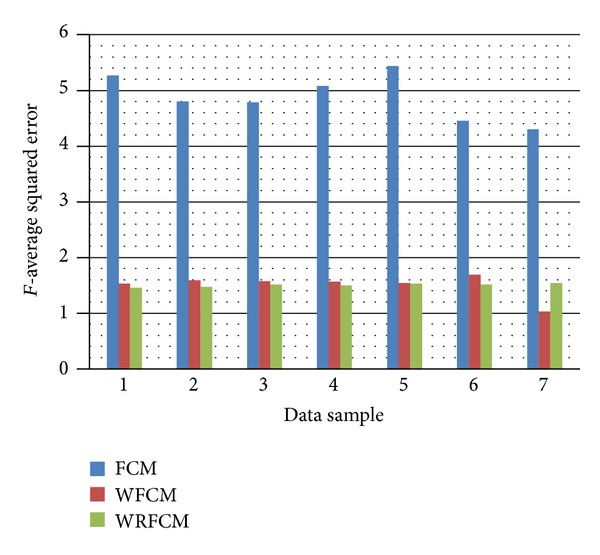
The average squared error (*F*) of the FCM algorithm, the WFCM algorithm, and the WRFCM algorithm on the seven data samples.

**Figure 7:**
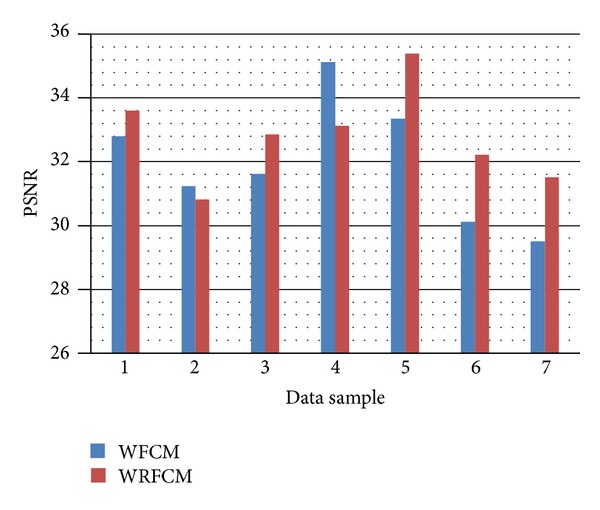
The PSNR of the WFCM algorithm, and the WRFCM algorithm on the seven data samples.

However, the visual acceptance is not enough thus we will use the *F*-average squared error and PSNR as segmentation evaluation metrics to evaluate the performance of the proposed Wavelet Relational Fuzzy *C*-Means (WRFCM) versus the conventional Fuzzy *C*-Means algorithm (FCM) and the Wavelet Fuzzy *C*-Means algorithm (WFCM). The results are plotted in Figures 6 and 7 and summarized in Tables 2 and 3.

From Table 2, we notice that all the results of the proposed algorithm are better than those of the FCM, while the results are better in 6 data samples than the WFCM (85.7% of the samples). The bold numbers in Table 2 represent the improvement caused by the proposed algorithm (WRFCM) over the WFCM algorithm. In the unimproved case, which is sample 7, the *F* error increases by (49.74%), while in the improved cases, the *F* error decreased down to (10.51%) in case 6.

From Table 3, we notice that the results are better in 5 data samples than the WFCM (71.4% of the samples). The bold numbers in Table 3 represent the improvement caused by the proposed algorithm (WRFCM) over the WFCM algorithm. In the unimproved cases, which are samples 2 and 4, the PSNR decreases by 1.327% and 5.65%, respectively. While in the improved cases, the PSNR increased up to (6.964%) in case 6.

We can also observe that in the first five data samples, which are data for the human leukemia and human blood lymphocytes, where the problems of ghost (weak) spots and noisy background exist, the proposed WRFCM algorithm, compared to the FCM algorithm and the WFCM algorithm, succeeded in identifying weak spots in all data samples. The reason behind the unimproved cases is that they suffered from noise that is why we will investigate the effect of removing the noise before segmentation in Section 6.

**Figure 8:**
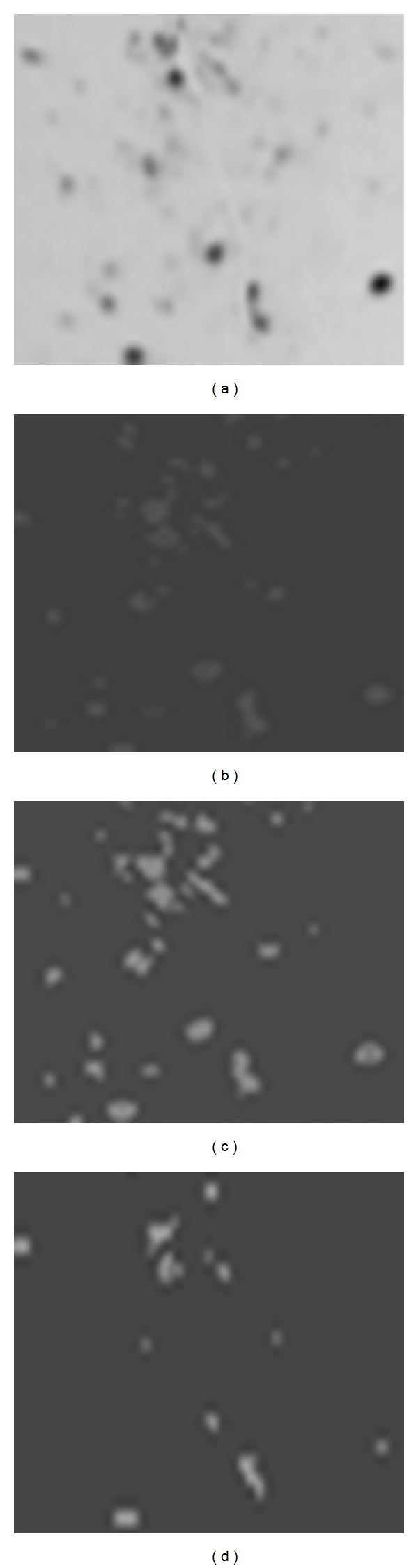
A close look at the 2D Gel electrophoresis image of the first sample of patient-human leukemias: (a) original image, (b) gradient image after applying FCM, (c) gradient image after applying WFCM, and (d) gradient image after WRFCM segmentation algorithms.

Figure 8 shows a close look at the left corner region of the image after applying the three segmentation algorithms. The figure proves the previous discussion on the effectiveness of the proposed algorithm and its ability to handle the oversegmentation error. We can also see that the parts that contain ghost spots (the parts that are found in light gray in the image background) were not considered as spot as they were get rid off through the wavelet application phase.

## 6. Applying Wavelet Denoising

In this section, we investigate the effect of performing denoising on the 2D gel images before segmenting the image using the proposed segmentation algorithm (WRFCM). We have chosen the orthogonal wavelet denoising, with its parameters adjusted using the genetic algorithm experimentally.

The orthogonal wavelet function has 4 parameters.

1. *The threshold selection rule* which can be “heursure” which is a heuristic variant of the Stein' Unbiased Risk Estimation (SURE) or minimax thresholding which uses a fixed threshold chosen to yield minimax performance for mean square error against an ideal procedure.
2. *The type of thresholding* which can be soft or hard thresholding.
3. *The multiplicative threshold rescaling* which can be no rescaling, rescaling using a single estimation of level noise based on first-level coefficients or rescaling using level-dependent estimation of level noise.
4. *The wavelet decomposition level*.

We used the genetic algorithm to find the best values of these parameters using the PSNR as the fitness function. The parameters obtained by the genetic algorithm were “heursure” as the threshold selection rule, hard thresholding, rescaling using level-dependent estimation of level noise, and decomposition level = 2.

We will compare the results of applying the proposed segmentation algorithm (WRFCM) with and without denoising performed before it. We intend to prove that the denoising can improve the segmentation results of these images.

**Figure 9:**
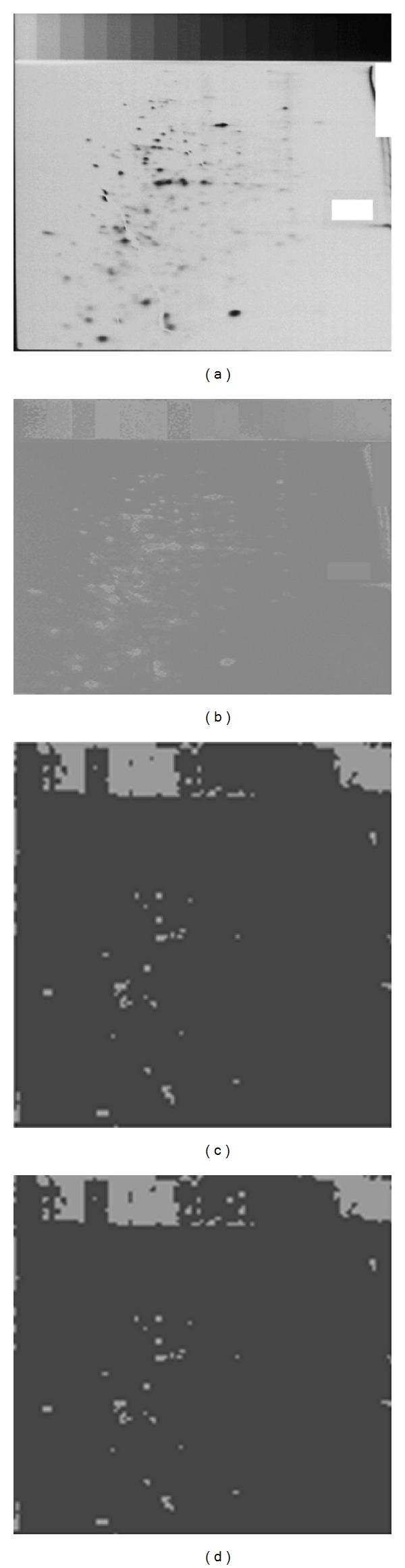
2D gel electrophoresis image of the first sample of patient-human leukemias: (a) original image, (b) gradient image, (c) gradient image after applying proposed WRFCM without denoising, and (d) gradient image after applying proposed WRFCM with denoising.

The parameters of the WRFCM used in this section are number of clusters (*C*) = 4 and *β* = 19. We measured the *F* average squared error and the PSNR of the images with and without denoising before being segmented.  Figure 9 shows applying the proposed WRFCM with and without denoising on one of the 7 data samples (the first sample of patient-human leukemias).

By visual inspection, we can observe that the problem of noisy background which leads to misclassified pixels had been overcome. In the resulted segmented image, after using denoising, many pixels which had been defined as “spot pixels” disappear. Only pixels which belong to the shape of spots had been classified as spot pixels. So, we can conclude that discarding noise from the 2D gel images is necessary for the accuracy of segmentation and the quantification step.

**Figure 10:**
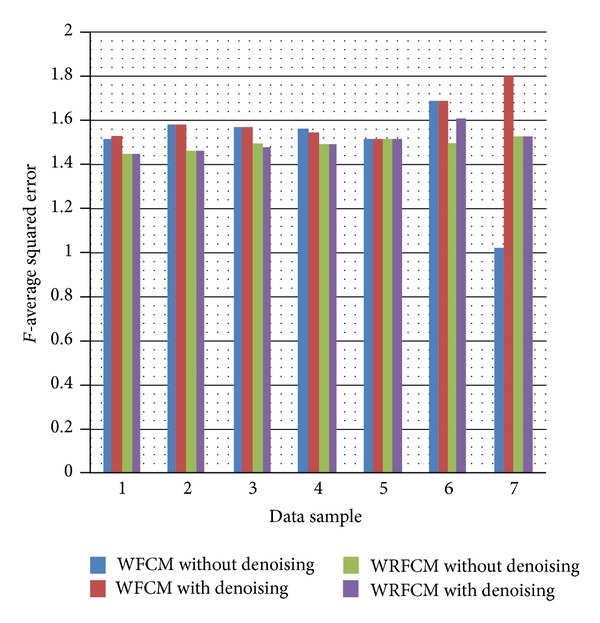
The average squared error (*F*) of the WFCM and WRFCM algorithms with and without denoising on the seven data samples.

**Figure 11:**
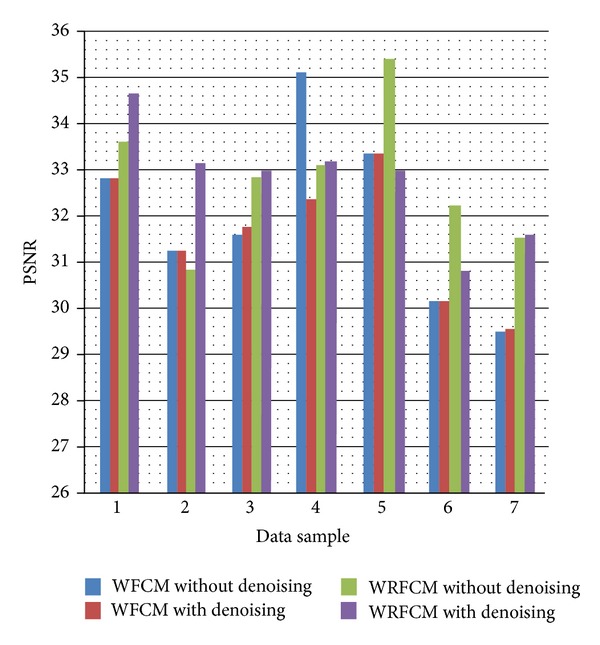
The PSNR of the WFCM and WRFCM algorithms with and without denoising on the seven data samples.

Then, we will use the *F* and PSNR evaluation errors (previously presented) to evaluate the performance of the (WFCM) algorithm without denoising versus (WRFCM) with and without denoising as shown in Tables 4 and 5 and Figures 10 and 11. N.B.: Samples in Tables 4 and 5 are the same and in the same order as in Tables 2 and 3.

The improvement in the *F* quality metric (which evaluates the oversegmentation) is poor in the resulted images after performing the denoising step before the segmentation but still this is an improvement. In the worst case which is case 6, the error increases by 6.895% while in the best three cases which are 3, 4, and 5, the error decreases by (1.352%), (0.034%), and (0.014%), respectively.

The improvement in the PSNR quality metric is also poor in the resulted images after performing the denoising step before the segmentation but still this is an improvement. In the worst case which is case 5, the PSNR decreases by (6.827%) while in the best case which is 2, the PSNR increases by (7.441%).

We can also observe that in the first five data samples, which are data for the human leukemia and human blood lymphocytes, where exist the problems of ghost (weak) spots and noisy background, the application of denoising technique before the proposed algorithm, compared to the WRFCM algorithm without denoising, succeeded in reducing the problem of oversegmentation and identifying weak spots as in all data samples.

## 7. Conclusions

The Fuzzy *C*-Means algorithm has the advantages of producing high quality segmentation compared to the other available algorithms. Our work in this paper was based on the Fuzzy *C*-Means algorithm by adding the notion of fuzzy relations and wavelets to it so as to enhance its performance especially in the area of 2D gel images. The parameters of the proposed algorithm, which are the number of clusters and the degree of closeness, were chosen experimentally.

We conducted experiments by applying the conventional (FCM) algorithm, the Wavelet Fuzzy *C*-Means algorithm, and the proposed algorithm (WRFCM) on 2D gel images acquired from the following:

1. Human leukemias (Eric Lester, Peter Lemkin),
2. HL-60 cell lines (Eric Lester, Peter Lemkin),
3. Fetal-alcohol-syndrome- (FAS-) serum (James Myrick, Mary Robinson, Peter Lemkin).

We compared the results of the algorithms both visually and numerically (using the *F* error and the PSNR quality metric). From these results we concluded that the proposed algorithm (WRFCM) surpasses the two other algorithms in most of the test cases under study in both criteria of comparison.

We also applied the wavelet denoising before the proposed algorithm and compared it to the results without denoising and the results of the Fuzzy *C*-Means with and without denoising to investigate the effect of denoising on the quality of the segmentation. From the results, we found out that the denoising enhanced the algorithm greatly.

## Figures and Tables

**Algorithm 1:**
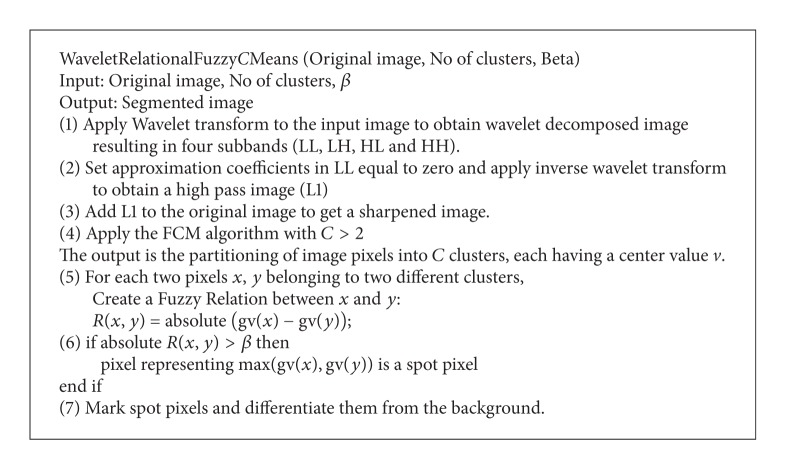
Algorithm WRFCM.

**Table 1:** *F* values at different values of WRFCM parameters *C* and *β* on sample of patient-human leukemia.

Test case no.	RFCM parameters	*F*	Time (sec.)
1	*C* = 4, *β* = 20	1.456501	79.84
2	*C* = 6, *β* = 20	1.454982	199.23
3	*C* = 8, *β* = 20	1.444162	210.29
4	*C* = 10, *β* = 20	1.428890	228.86
5	*C* = 4, *β* = 19	1.456453	88.89
6	*C* = 4, *β* = 21	1.456501	92.09
7	***C* = 4, ** **β** = 22	**1.485222**	**93.19**
8	*C* = 4, *β* = 23	1.456928	96.18
9	*C* = 4, *β* = 24	1.457071	90.82
10	*C* = 4, *β* = 25	1.457357	88.09

**Table 2:** The average squared error (*F*) of the FCM algorithm, the WFCM algorithm, and the WRFCM algorithm on the seven data samples.

Sample no.	*F* (FCM)	*F* (WFCM)	*F* (WRFCM)	Improvement %
Human leukemia				
1	**5.2670**	**1.5336**	**1.4564**	**5.033907**
2	**4.8006**	**1.5889**	**1.4747**	**7.187362**
3	**4.7877**	**1.5762**	**1.5093**	**4.244385**

Human blood lymphocytes				
4	**5.0801**	**1.5655**	**1.4957**	**4.458639**
5	**5.4390**	**1.5341**	**1.5299**	**0.273776**

Fetal alcohol syndrome				
6	**4.4498**	**1.6903**	**1.5126**	**10.51293**
7	4.3001	1.0270	1.5379	−49.7468

**Table 3:** The PSNR of the WFCM algorithm, and the WRFCM algorithm on the seven data samples.

Sample no.	PSNR (WFCM)	PSNR (WRFCM)	Improvement %
Human leukemia			
1	**32.795091**	**33.599365**	**2.452421**
2	31.231596	30.817014	−1.327444
3	**31.623994**	**32.855681**	**3.894786**

Human blood lymphocytes			
4	35.118125	33.130932	−5.658596
5	**33.354534**	**35.391646**	**6.107451**

Fetal alcohol syndrome			
6	**30.116019**	**32.213339**	**6.964134**
7	**29.504009**	**31.513995**	**6.812586**

**Table 4:** The average squared error (*F*) of the WFCM and the WRFCM algorithm with and without denoising on the seven data samples.

Sample no.	*F* (WRFCM without denoising)	*F* (WRFCM with denoising)	Improvement %	*F* (WFCM without denoising)	*F* (WFCM with denoising)	Improvement %
Human leukemia						
1	1.456453	1.456405	0.003295	**1.533642**	**1.5383**	**−0.3088**
2	1.474703	1.474900	−0.013358	**1.588977**	**1.5912**	**−0.1439**
3	1.509377	1.488963	1.352478	**1.576237**	**1.5781**	**−0.1225**

Human blood lymphocytes						
4	1.495710	1.495196	0.034364	**1.565566**	**1.5621**	**0.2178**
5	1.529938	1.529718	0.014379	**1.534169**	**1.5336**	**0.0343**

Fetal alcohol syndrome						
6	1.512667	1.616971	−6.895370	**1.690317**	**1.6887**	**0.0954**
7	1.537931	1.537763	0.010923	**1.027005**	**1.7973**	**−75.0117**

Average			−0.784755	Average		**−10.7485**

**Table 5:** The PSNR of the WFCM and the WRFCM algorithm with and without denoising algorithm with and without denoising on the seven data samples.

Sample no.	PSNR (WRFCM without denoising)	PSNR (WRFCM with denoising)	Improvement %	PSNR (WFCM without denoising)	PSNR (WFCM with denoising)	Improvement %
Human leukemia						
1	33.599365	34.649476	3.125389423	**32.795091**	**32.797204**	**0.006443**
2	30.817014	33.110287	7.44158081	**31.231596**	**31.241120**	**0.030495**
3	32.855681	32.955918	0.305082704	**31.623994**	**31.765727**	**0.448182**

Human blood lymphocytes						
4	33.130932	33.153382	0.067761	**35.118125**	**32.373676**	**−7.81491**
5	35.391646	32.975295	−6.827461	**33.354534**	**33.361656**	**0.021352**

Fetal alcohol syndrome						
6	32.213339	30.802307	−4.380272	**30.116019**	**30.124187**	**0.027122**
7	31.513995	31.558443	0.141042	**29.504009**	**29.511908**	**0.026773**

Average			−0.018125	Average		**−1.03636**
